# Urban-rural disparities in child linear growth: a decomposition analysis of digital, physical, and socioeconomic environments in seven least-developed countries

**DOI:** 10.7189/jogh.16.04158

**Published:** 2026-07-01

**Authors:** Zhixin Liu, Rizhen Wang, Xiyu Zhang, Qunhong Wu

**Affiliations:** 1 School of Public Health, Peking University, Beijing, China; 2 School of Health Management, Harbin Medical University, Harbin, China

## Abstract

**Background:**

Child linear growth lags behind in rural areas compared to urban areas in least developed countries (LDCs). The extent to which household environments – digital, physical, and socioeconomic – are associated with this urban-rural gap remains unclear. We quantified how these household environments contribute to the urban-rural difference in linear growth among children.

**Methods:**

We pooled cross-sectional data from the demographic and health surveys conducted between 2015 and 2024 in seven LDCs. The analysis included children aged 0–59 months. Child linear growth was measured using the height-for-age Z-score (HAZ), computed from standard anthropometric measurements using the World Health Organization Child Growth Standards. We estimated the urban-rural difference in HAZ using hierarchical regression models and decomposed the mean gap into contributions from household digital, physical, and socioeconomic environments using Blinder-Oaxaca decomposition.

**Results:**

The pooled sample included 59 219 children (28.0% urban and 72.0% rural). The urban-rural difference in HAZ decreased from −0.29 (95% confidence interval (CI) = −0.32, −0.27) to −0.02 (95% CI = −0.05, 0.01) after adjustment for demographic characteristics and household environments. Blinder-Oaxaca decomposition indicated that over 95% of the pooled urban-rural HAZ gap was explained by measured household characteristics. The largest contributions were from household wealth (richest *vs*. poorest percentage explained = 38.8%), maternal education (secondary or higher *vs*. none = 19.6%), digital access (17.4%), and daily maternal Internet use (10.2%). Physical-environment indicators were smaller and less consistent across countries, although improved sanitation contributed positively in the pooled analysis (percentage explained = 10.4%).

**Conclusions:**

The urban-rural disparity in child linear growth is largely explained by unequal distributions of socioeconomic, digital, and physical household resources.

Ending child malnutrition is a central ambition of Sustainable Development Goal 2.2. Yet progress in reducing growth faltering remains uneven, with the heaviest burdens concentrated in the world’s least developed countries (LDCs) [1]. LDCs face distinctive structural constraints – persistently low income, weak human assets, and high economic fragility – further exacerbated by the COVID-19 pandemic [2]. In response, the United Nations (UN) Doha Programme of Action (2022–2031) aims to support LDCs in overcoming these barriers [3]. This agenda is directly relevant to child linear growth, as global targets are unlikely to be met without faster progress in this priority group.

Within LDCs, child growth outcomes are unequally distributed and a persistent urban-rural gap in under-five stunting has been widely documented [4]. To characterise this disparity, we extracted the most recent Children’s Fund-reported stunting prevalence by residence for 24 UN-classified LDCs (surveys conducted in 2009–2023) and pooled estimates using an inverse-variance-weighted random-effects model. The pooled rural prevalence was 39.8% (95% confidence interval (CI) = 39.4, 40.2), compared with 25.1% (95% CI = 24.5, 25.7) in urban areas (Appendix S1 in the **Online Supplementary Document**) [5]. Because stunting reflects chronic deficits in linear growth among children, these differences indicate substantial inequality in children’s attained height-for-age across settings. The consequences of growth faltering are profound because they impair cognitive development, reduce educational attainment, and diminish adult productivity [6,7]. This creates intergenerational cycles of poverty that hinder national progress and underscore that national-level strategies may fail unless they address the unique factors of rural inequality.

According to UNICEF’s 2020 conceptual framework on the determinants of child nutrition, the household environment is the proximal setting in which diets and caregiving practices are enacted [8]. Although households are embedded within broader community and structural contexts, such as local food systems, prices, and health services, the household remains the immediate locus through which these upstream conditions are accessed and translated into child-relevant resources [9,10]. In LDCs, rural residence is consistently associated with systematic disadvantages in these resources. Decomposing the household environment, therefore, allows us to quantify the extent to which the urban-rural height-for-age Z-score (HAZ) gap is associated with specific resource inequalities, rather than with residence as a fixed geographic label. While existing literature documents these disparities, it has focused primarily on socioeconomic [11–14] and physical infrastructure [15–19], often leaving a substantial portion of the gap unexplained [19–22]. Furthermore, evidence from LDCs remains limited and largely derives from a small number of single-country studies (*e.g.* Malawi, Rwanda, and Ethiopia) [22–24], which constrains insights into cross-national regularities and differences. Crucially, the digital environment remains a systematically overlooked correlate [25]. Digital connectivity offers potential avenues for nutritional support: facilitating access to health information, enabling connections with health systems, and supporting financial inclusion to buffer shocks [26–29]. These factors likely reflect a unique layer of opportunity structure distinct from wealth or education. Accordingly, examining the digital environment alongside socioeconomic and physical environments provides a more complete account of the urban–rural gradient in household resources and yields more actionable evidence for multi-sectoral policy responses.

To address these gaps, we used Demographic and Health Survey (DHS) data from seven LDCs to examine the relative contributions of household socioeconomic, physical, and digital environments in explaining the urban-rural disparities in child linear growth.

## METHODS

### Study design and participants

We conducted a multi-country, cross-sectional pooled analysis using the most recent DHS conducted between 2015 and 2024. This study is reported in accordance with the STROBE guidelines. Furthermore, we followed the GRABDROP guidance for secondary analyses of large public repositories (Table S1 in the **Online Supplementary Document**) [30]. We used standard DHS data (phases seven and eight), which employ a two-stage stratified cluster design to produce nationally representative estimates [31].

Country selection was conducted through a three-step process. First, we restricted our sample to nations classified as LDCs by the UN at the time of the survey. These countries share structural constraints as defined by the UN Committee for Development Policy – low income (gross national income per capita<USD 1088), high structural vulnerability (economic and environmental vulnerability index >36), and deficits in human assets (including child stunting as a defining indicator). All included countries retained LDC status throughout the study period, indicating persistent development constraints. Second, the inclusion period was restricted to surveys conducted from 2015 onwards to ensure the availability of digital questionnaire modules essential to our research objectives. Third, to ensure data reliability, countries with more than 20% missing data for key anthropometric variables were excluded. Seven countries met these criteria: Benin (2017–2018), Ethiopia (2016), Haiti (2016–2017), Mali (2023–2024), Myanmar (2015–2016), Timor-Leste (2016), and Zambia (2018–2019).

Eligible participants were children aged 0–59 months. We excluded children with missing or implausible anthropometric measurements (HAZ<−6 standard deviations (SD) or >6 SD).

### Assessments

#### Outcome variable

Child linear growth was measured using HAZ, based on standard DHS anthropometric protocols [31] and calculated using the World Health Organization (WHO) Child Growth Standards [32]. We modelled HAZ as continuous to preserve distributional information [33] and to support decomposition of mean urban-rural differences; stunting (HAZ<−2 SD) was examined in sensitivity analyses.

#### Exposure variable

We classified residence as urban *vs*. rural using each country’s official definition. Although no single, standardised global definition exists, the UN Statistics Division recommends basing this classification primarily on measures of population density. Other criteria may include the percentage of the population engaged in agriculture, the availability of infrastructure (*e.g.* electricity and piped water), or access to services such as health care, schools, and transportation.

#### Explanatory variables

We grouped explanatory variables into three domains of the household environment – digital, physical, and socioeconomic. We assessed digital environment using three factors representing access, usage, and electronic financial account [25]. Access was defined as household possession of a mobile phone or computer (yes/no). Usage was measured by the maternal Internet use frequency, categorised as ‘never’ (reference), ‘at least weekly,’ or ‘almost daily.’ Electronic financial account was defined as the family having an electronic account for financial transactions (yes/no).

We assessed the physical environment using six factors. Three factors related to water, sanitation, and hygiene: drinking water (improved *vs*. unimproved) and sanitation facility (improved *vs*. unimproved) were categorised based on standard DHS and WHO/UNICEF joint monitoring programme definitions. Handwashing facility was defined as the presence of a fixed or mobile facility at the dwelling (present *vs*. absent). The remaining three factors assessed household infrastructure. Cooking fuel was defined based on WHO recommendations (clean *vs*. solid fuels) [34]. Availability of electricity was a binary variable (yes *vs*. no), and overcrowding was defined as ≥3 people per sleeping room.

The socioeconomic environment was operationalised using three indicators. First, we measured household wealth using a composite index derived from principal component analysis (PCA), based on a harmonised set of household economic assets and living-standard indicators, including durable goods (radio, television, refrigerator, bicycle, motorcycle, and car/truck), housing quality (floor, wall, and roof materials), land ownership, and livestock ownership. To enhance cross-country comparability, only variables consistently available across all included surveys were retained in the index construction. The resulting wealth score was classified into country-specific quintiles (poorest to richest), representing relative socioeconomic position within each country rather than absolute wealth comparability across countries. The other two indicators were maternal education and paternal education, each categorised as none, primary, or secondary and above.

### Covariates

Covariates were guided by the UNICEF conceptual framework for child malnutrition [35], which distinguishes between underlying household determinants and immediate proximate determinants. To prevent over-adjustment bias, we adjusted our primary models only for exogenous demographic and biological confounders. These included child age (categorised as 0–11, 12–23, 24–35, 36–47, and 48–59 months), child sex (male *vs*. female), birth order (1, 2-4, and ≥5), and sex of household head (female *vs*. male).

Variables potentially on the causal pathway were treated as mediators and excluded from primary models (size at birth, minimum dietary diversity, barriers to health care access), but included in extended models as sensitivity analyses.

### Statistical analysis

We examined differences in the distribution of household environments and other covariates by residence using χ^2^ tests for categorical variables. We visualised HAZ distributions by residence using kernel density plots for pooled and country-specific samples.

To estimate population-weighted pooled associations and avoid over-weighting smaller countries with large sample sizes, we rescaled DHS sampling weights using N_pop_/n_sample_, where N_pop_ represents the national under-five population size (World Population Prospects, survey year) and n_sample_ is the survey sample size.

We addressed missing data using multiple imputation by chained equations for variables with incomplete information, specifically father’s education (10.8%), housing density (0.2%), maternal Internet use (7.6%), size at birth (12.6%), and health care access issues (0.5%). The imputation models incorporated all variables used in the main analysis, including HAZ, residence, environmental exposures, and all covariates [36]. We generated 20 imputed data sets per country, employing logistic and ordered logistic regressions for binary and ordinal variables, respectively. To account for the complex survey design, survey weights were included in the imputation process, though primary sampling unit (PSU) indicators were omitted to avoid non-convergence and collinearity.

All regression and decomposition analyses accounted for complex survey design (weights and clustering). We fitted hierarchical ordinary least squares (OLS) models: model 1 (included residence and country fixed effects only), model 2 (further adjusted for demographic characteristics), and model 3 (further adjusted for household-environment indicators). Multicollinearity was assessed using variance inflation factors (maximum <3). Effect modification by child sex was tested using interaction terms (sex × residence and sex × domain indicators), evaluated with Wald and joint Wald tests. We applied the Blinder-Oaxaca decomposition to partition the mean urban-rural HAZ gap into explained (endowment) and unexplained components [37]. To reduce index-number sensitivity, we used pooled coefficients with a group indicator as the reference [38]. We conducted a pooled seven-country decomposition (treating country fixed effects as nuisance controls) and country-specific decompositions to assess heterogeneity. We applied Rubin’s rules to combine estimates and uncertainty across imputations for regression coefficients and absolute decomposition contributions. The percentage explained by each domain was reported as a descriptive point estimate derived from pooled absolute components [39].

We conducted six pre-specified sensitivity analyses: Fairlie decomposition for binary stunting (HAZ<−2 SD) [40]; use of original DHS weights (no population reweighting); complete-case analysis; substitution of the harmonised wealth index with DHS wealth quintiles; exclusion of infrastructure indicators potentially overlapping with residence definitions; and addition of proximate determinants (barriers to care, size at birth, and minimum dietary diversity), with dietary diversity analyses restricted to children aged 6–23 months.

We performed all analyses using Stata, version 17.0 (StataCorp, College Station, Texas, USA). Two-sided *P*-value <0.05 indicated statistical significance.

## RESULTS

### Sample characteristics

The pooled sample included 59 219 children aged 0–59 months, of whom 28.0% (n = 16 581) lived in urban areas and 72.0% (n = 42 638) in rural areas (**Table 1**). Child sex and age distributions were balanced by residence, but rural children were disproportionately concentrated in the poorest wealth quintile (29.0% *vs*. 8.6%), and half of rural mothers (50.5%) had no formal education compared with 29.2% of urban mothers. Infrastructure deprivation was more common in rural households, particularly a lack of electricity (74.6% *vs*. 29.8%) and unimproved sanitation (64.9% *vs*. 32.7%). A marked digital divide was also evident: most rural mothers reported never using the internet (85.4%), and rural households were less likely to use electronic financial electronic financial accounts (14.2% *vs*. 39.2%).

**Table 1:** Characteristics of children aged 0–59 mo by urban-rural residence across seven least developed countries, 2015–2024

Variable	Overall (n = 59 219)	Urban (n = 16 581)	Rural (n = 42 638)	*P*-value
**Digital environment**				
Digital access				<0.001
*No*	11 561 (19.5)	1110 (6.7)	10 451 (24.5)	
*Yes*	47 658 (80.5)	15 471 (93.3)	32 187 (75.5)	
Maternal Internet use				<0.001
*Never*	48 682 (82.2)	12 263 (74.0)	36 419 (85.4)	
*Weekly*	2798 (4.7)	1337 (8.1)	1461 (3.4)	
*Daily*	3259 (5.5)	2061 (12.4)	1198 (2.8)	
*Missing*	4480 (7.6)	920 (5.5)	3560 (8.3)	
Electronic financial account				<0.001
*No*	46 679 (78.8)	10 082 (60.8)	36 597 (85.8)	
*Yes*	12 540 (21.2)	6499 (39.2)	6041 (14.2)	
**Physical environment**				
Cooking fuel				<0.001
*Solid*	56 613 (95.6)	14 620 (88.2)	41 993 (98.5)	
*Clean*	2606 (4.4)	1961 (11.8)	645 (1.5)	
Drinking water				<0.001
*Unimproved*	18 839 (31.8)	3062 (18.5)	15 777 (37.0)	
*Improved*	40 380 (68.2)	13 519 (81.5)	26 861 (63.0)	
Sanitation facility				<0.001
*Unimproved*	33 112 (55.9)	5427 (32.7)	27 685 (64.9)	
*Improved*	26 107 (44.1)	11 154 (67.3)	14 953 (35.1)	
Handwashing				<0.001
*Absent*	19 263 (32.5)	4644 (28.0)	14 619 (34.3)	
*Present*	39 956 (67.5)	11 937 (72.0)	28 019 (65.7)	
Availability of electricity				<0.001
*No*	36 736 (62.0)	4934 (29.8)	31 802 (74.6)	
*Yes*	22 483 (38.0)	11 647 (70.2)	10 836 (25.4)	
Housing density				<0.001
*Overcrowded*	35 955 (60.7)	9310 (56.1)	26 645 (62.5)	
*Not overcrowded*	23 141 (39.1)	7259 (43.8)	15 882 (37.2)	
*Missing*	123 (0.2)	12 (0.1)	111 (0.3)	
**Socioeconomic environment**				
Household wealth				<0.001
*Poorest*	13 795 (23.3)	1419 (8.6)	12 376 (29.0)	
*Poorer*	13 241 (22.4)	1220 (7.4)	12 021 (28.2)	
*Middle*	11 076 (18.7)	2423 (14.6)	8653 (20.3)	
*Richer*	11 356 (19.2)	4611 (27.8)	6745 (15.8)	
*Richest*	9751 (16.5)	6908 (41.7)	2843 (6.7)	
Mother’s education				<0.001
*None*	26 380 (44.5)	4837 (29.2)	21 543 (50.5)	
*Primary*	16 669 (28.1)	3852 (23.2)	12 817 (30.1)	
*Secondary and more*	16 170 (27.3)	7892 (47.6)	8278 (19.4)	
Father’s education				<0.001
*None*	22 206 (37.5)	3743 (22.6)	18 463 (43.3)	
*Primary*	13 416 (22.7)	2714 (16.4)	10 702 (25.1)	
*Secondary and more*	17 223 (29.1)	7785 (47.0)	9438 (22.1)	
*Missing*	6374 (10.8)	2339 (14.1)	4035 (9.5)	
**Covariates**				
Child sex				0.74
*Male*	29 944 (50.6)	8366 (50.5)	21 578 (50.6)	
*Female*	29 275 (49.4)	8215 (49.5)	21 060 (49.4)	
Child’s age in months				0.163
*0–11*	12 806 (21.6)	3645 (22.0)	9161 (21.5)	
*12–23*	12 093 (20.4)	3454 (20.8)	8639 (20.3)	
*24–35*	11 616 (19.6)	3247 (19.6)	8369 (19.6)	
*36–47*	11 500 (19.4)	3157 (19.0)	8343 (19.6)	
*48–59*	11 204 (18.9)	3078 (18.6)	8126 (19.1)	
Birth order				<0.001
*1*	13 450 (22.7)	4513 (27.2)	8937 (21.0)	
*2–4*	28 377 (47.9)	8665 (52.3)	19 712 (46.2)	
*≥5*	17 392 (29.4)	3403 (20.5)	13 989 (32.8)	
Sex of household head				<0.001
*Male*	48 753 (82.3)	13 101 (79.0)	35 652 (83.6)	
*Female*	10 466 (17.7)	3480 (21.0)	6986 (16.4)	
Size at birth				<0.001
*Average*	28 680 (48.4)	8677 (52.3)	20 003 (46.9)	
*Small*	8563 (14.5)	2131 (12.9)	6432 (15.1)	
*Large*	14 518 (24.5)	4203 (25.3)	10 315 (24.2)	
*Missing*	7458 (12.6)	1570 (9.5)	5888 (13.8)	
Problems accessing care				<0.001
*No*	22 672 (38.3)	8527 (51.4)	14 145 (33.2)	
*Yes*	36 280 (61.3)	8001 (48.3)	28 279 (66.3)	
*Missing*	267 (0.5)	53 (0.3)	214 (0.5)	

### Disparities in mean HAZ

**Figure 1:**
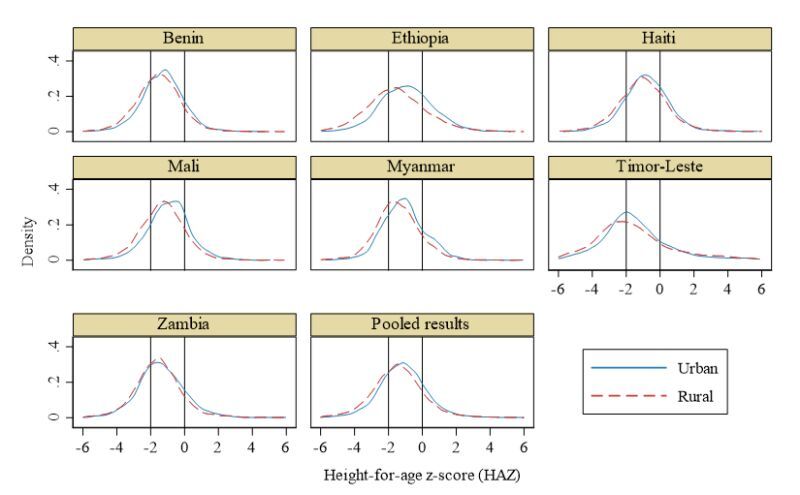
Overall and country-specific urban-rural disparities in the distribution of child height-for-age Z-scores (HAZ), n (%).

Across all seven countries, rural residence was associated with poorer linear growth, measured by child HAZ. Mean (x̄) HAZ was consistently lower among rural children than urban children, with the largest gap in Ethiopia (rural x̄ = –1.47; 95% CI = –1.51, –1.43 *vs*. urban x̄ = –0.90; 95% CI = –0.98, –0.83). Even in settings with high overall stunting burden, such as Timor-Leste and Zambia, a rural disadvantage persisted (**Figure 1**).

### Factors associated with HAZ

We estimated associations with HAZ using hierarchical OLS regression models (**Table 2**). In the unadjusted model, rural residence was associated with lower HAZ (β = –0.29; 95% CI = –0.32, –0.27). This association remained similar after adjustment for demographic characteristics (β = –0.28; 95% CI = –0.31, –0.26). In the fully adjusted model, additional inclusion of household environmental domains attenuated the rural-urban association to a small and statistically non-significant estimate (β = –0.02; 95% CI = –0.05, 0.01). A socioeconomic gradient was evident. Children in the richest wealth quintile had higher HAZ than those in the poorest quintile (β = 0.28; 95% CI = 0.22, 0.33), and maternal secondary education was positively associated with HAZ (β = 0.11; 95% CI = 0.07, 0.15). Digital inclusion indicators were associated with HAZ, including daily maternal Internet use (β = 0.28; 95% CI = 0.22, 0.33), household device access (β = 0.17; 95% CI = 0.14, 0.21), and electronic financial account use (β = 0.04; 95% CI = 0.01, 0.08). Several physical infrastructure indicators also showed positive associations, including clean cooking fuel (β = 0.14; 95% CI = 0.08, 0.20), non-overcrowded housing (β = 0.06; 95% CI = 0.03, 0.08), electricity (β = 0.03; 95% CI = 0.00, 0.07), and improved sanitation (β = 0.03; 95% CI = 0.01, 0.06), whereas drinking water source and handwashing facilities were not statistically associated.

**Table 2:** Hierarchical OLS models of associations between household environments and child height-for-age Z-score (HAZ)*

Variables	Model 1 (unadjusted), β (95% CI)	Model 2 (demographic-adjusted), β (95% CI)	Model 3 (fully adjusted), β (95% CI)
Residence			
*Urban*	Ref.	Ref.	Ref.
*Rural*	−0.29 (−0.32, −0.27)	−0.28 (−0.31, −0.26)	−0.02 (−0.05, 0.01)
Child’s sex			
*Male*		Ref.	Ref.
*Female*		0.14 (0.12, 0.17)	0.15 (0.12, 0.17)
Child age in months			
*0–11*		Ref.	Ref.
*12–23*		−0.76 (−0.80, −0.72)	−0.76 (−0.79, −0.72)
*24–35*		−0.96 (−1.00, −0.92)	−0.95 (−0.99, −0.91)
*36–47*		−0.88 (−0.92, −0.84)	−0.87 (−0.90, −0.83)
*48–59*		−0.69 (−0.73, −0.65)	−0.67 (−0.71, −0.64)
Birth order			
*1*		Ref.	Ref.
*2–4*		0.01 (−0.02, 0.04)	0.07 (0.04, 0.10)
*≥5*		−0.05 (−0.08, −0.01)	0.08 (0.05, 0.12)
Sex of household head			
*Male*		Ref.	Ref.
*Female*		0.03 (−0.01, 0.06)	0.04 (0.01, 0.07)
Digital access			
*No*			Ref.
*Yes*			0.17 (0.14, 0.21)
Maternal Internet use			
*Never*			Ref.
*Weekly*			0.19 (0.13, 0.25)
*Daily*			0.28 (0.22, 0.33)
Electronic financial account			
*No*			Ref.
*Yes*			0.04 (0.01, 0.08)
Cooking fuel			
*Solid*			Ref.
*Clean*			0.14 (0.08, 0.20)
Drinking water			
*Unimproved*			Ref.
*Improved*			0.01 (–0.02, 0.03)
Sanitation facility			
*Unimproved*			Ref.
*Improved*			0.03 (0.01, 0.06)
Handwashing			
*Absent*			Ref.
*Present*			0.02 (–0.01, 0.05)
Availability of electricity			
*No*			Ref.
*Yes*			0.03 (0.00, 0.07)
Housing density			
*Overcrowded*			Ref.
*Not overcrowded*			0.06 (0.03, 0.08)
Household wealth			
*Poorest*			Ref.
*Poorer*			0.04 (0.00, 0.08)
*Middle*			0.08 (0.04, 0.12)
*Richer*			0.13 (0.08, 0.17)
*Richest*			0.28 (0.22, 0.33)
Maternal education			
*None*			Ref.
*Primary*			0.03 (–0.01, 0.06)
*Secondary and more*			0.11 (0.07, 0.15)
Paternal education			
*None*			Ref.
*Primary*			0.06 (0.03, 0.10)
*Secondary and more*			0.07 (0.03, 0.11)

CI – confidence interval, ref – reference

*β are from ordinary least squares (OLS) regressions with 95% CI. All models include country fixed effects and account for complex survey design using rescaled DHS sampling weights and robust standard errors clustered at the primary sampling unit (PSU) level. Estimates are based on multiply-imputed data and are combined using Rubin’s rules.

We assessed effect modification by child sex using interaction terms and joint Wald tests (Table S2 in the **Online Supplementary Document**). There was no evidence that associations differed by sex for residence (*P* = 0.788), physical (*P* = 0.392), and socioeconomic (*P* = 0.072) domains. On this basis, the pooled models were retained as primary analyses.

### Blinder-Oaxaca decomposition of the urban-rural HAZ gap

To quantify contributors to the urban-rural HAZ gap, we applied Blinder-Oaxaca decomposition (**Table 3**). The total predicted urban-rural gap in HAZ, defined as the predicted mean HAZ difference between urban and rural children, was 0.360 (95% CI = 0.279, 0.441) z-score units. Of this gap, 0.349 (95% CI = 0.280, 0.418) z-score units was explained by differences in observed characteristics. The unexplained component was 0.012 (95% CI = −0.074, 0.097) z-score units, representing the remaining portion of the HAZ gap not accounted for by the variables included in the decomposition model. The socioeconomic environment contributed most to the explained component (β = 0.222; 95% CI = 0.158, 0.287), accounting for 63.7%, with major contributors including wealth (richest *vs*. poorest β = 0.135; 38.8%), maternal secondary or higher education (β = 0.068; 19.6%), and paternal secondary or higher education (β = 0.051; 14.6%). The digital environment contributed 28.1% (β = 0.098; 95% CI = 0.062, 0.134), largely through device access (17.4%) and maternal Internet use (weekly 4.1% and daily 10.2%). The physical environment accounted for 20.3% (β = 0.071; 95% CI = 0.017, 0.125), with notable contributions from improved sanitation (10.4%) and lower housing crowding (4.5%). In our pooled analysis, country fixed effects serve as a compositional control to account for their negative explained contribution, not as a core explanatory factor.

**Table 3:** Blinder-Oaxaca decomposition of the urban-rural gap in child height-for-age z-score (HAZ): pooled seven-country results*

Factor	β (95% CI)	*P*-value	Percentage explained, %
Digital environment	0.098 (0.062, 0.134)	<0.001	28.1
*Access (yes vs. no)*	0.061 (0.035, 0.087)	<0.001	17.4
*Maternal Internet use (weekly vs. never)*	0.014 (0.009, 0.020)	<0.001	4.1
*Maternal Internet use (daily vs. never)*	0.036 (0.024, 0.048)	<0.001	10.2
*Electronic financial account (yes vs. no)*	−0.013 (−0.041, 0.016)	0.385	−3.6
Physical environment	0.071 (0.017, 0.125)	0.010	20.3
*Cooking fuel (clean vs. solid)*	0.017 (−0.009, 0.043)	0.211	4.8
*Drinking water (improved vs. unimproved)*	−0.011 (−0.034, 0.011)	0.316	−3.3
*Sanitation facility (improved vs. unimproved)*	0.036 (0.011, 0.062)	0.005	10.4
*Handwashing (present vs. absent)*	−0.002 (−0.015, 0.011)	0.754	−0.6
*Availability of electricity (yes vs. no)*	0.016 (−0.030, 0.061)	0.503	4.5
*Housing density (not overcrowded vs. overcrowding)*	0.016 (0.007, 0.024)	<0.001	4.5
Socioeconomic environment	0.222 (0.158, 0.287)	<0.001	63.7
*Household wealth (poor vs. poorest)*	−0.024 (−0.047, −0.001)	0.038	−7.0
*Household wealth (middle vs. poorest)*	−0.006 (−0.011, −0.001)	0.016	−1.7
*Household wealth (richer vs. poorest)*	0.016 (0.006, 0.027)	0.003	4.7
*Household wealth (richest vs. poorest)*	0.135 (0.078, 0.192)	<0.001	38.8
*Maternal education (primary vs. none)*	–0.002 (–0.006, 0.003)	0.445	–0.5
*Maternal education (secondary + vs. none)*	0.068 (0.034, 0.102)	<0.001	19.6
*Paternal education (primary vs. none)*	−0.017 (−0.031, –0.002)	0.023	−4.8
*Paternal education (secondary + vs. none)*	0.051 (0.013, 0.088)	0.008	14.6
Covariates	−0.010 (−0.033, 0.013)	0.382	−2.9
*Child sex (female vs. male)*	0.000 (−0.003, 0.003)	0.841	0.1
*Child age (12–23 vs. 0–11 mo)*	−0.009 (−0.023, 0.005)	0.193	−2.6
*Child age (24–35 vs. 0–11 mo)*	0.002 (−0.013, 0.017)	0.800	0.6
*Child age (36–47 vs. 0–11 mo)*	0.003 (−0.011, 0.018)	0.666	0.9
*Child age (48–59 vs. 0–11 mo)*	0.020 (0.007, 0.032)	0.003	5.6
*Birth order (2–4 vs. 1)*	0.005 (0.000, 0.010)	0.033	1.6
*Birth order (≥5 vs 1)*	−0.026 (−0.043, −0.008)	0.004	−7.3
*Sex of household head (female vs. male)*	−0.006 (−0.012, 0.000)	0.055	−1.7
Country fixed effects	−0.032 (−0.058, −0.006)	0.015	−9.3
Total explained gap	0.349 (0.280, 0.418)	<0.001	100
Total unexplained gap	0.012 (−0.074, 0.097)	0.793	
Total predicted gap	0.360 (0.279, 0.441)	<0.001	

CI – confidence interval

*Country fixed effects are included as nuisance controls to absorb cross-country compositional heterogeneity; their contribution is interpreted as a structural adjustment term rather than an explanatory factor. Estimates are from multiply imputed data, pooled with Rubin’s rules; 95% confidence intervals reflect sampling and imputation uncertainty.

Country-specific decompositions showed variability in the explained component (*e.g.* 0.164 in Timor-Leste; 95% CI = 0.016, 0.313 *vs*. 0.511 in Ethiopia; 95% CI = 0.296, 0.726), with socioeconomic and digital domains generally contributing positively but with context-specific precision (Table S3 in the **Online Supplementary Document**).

### Sensitivity analyses

Across six sensitivity analyses, results were directionally consistent, including Fairlie decomposition for stunting, alternative weighting, complete-case analysis, use of the standard DHS wealth index, exclusion of residence-correlated infrastructure variables, and additional adjustment for proximal factors (Tables S4–S9 in the **Online Supplementary Document**).

## DISCUSSION

In this study, we assessed the relative contributions of household digital, physical, and socioeconomic environments to urban-rural differences in child linear growth across seven UN-classified LDCs. We found a clear rural disadvantage in HAZ, and this disparity was largely accounted for by differences in observed household resources in the decomposition framework. Overall, the full model explained 97% of the urban-rural HAZ gap, leaving only a small unexplained component. Within the explained component, socioeconomic conditions accounted for the largest share, but the digital environment also contributed substantially – exceeding that of the physical environment in the primary model.

Our finding of an urban-rural HAZ gap is consistent with evidence from multiple single-country studies [41]. The important roles of socioeconomic conditions and maternal education also align with prior multi-country and longitudinal research [14,23,42,43]. Our results add two key nuances. First, whereas earlier decomposition studies reported sizeable unexplained components (*e.g.*>60% in Ethiopia) [22], our model explained more than 90% of the gap, indicating that broader household-environment measurement can capture a larger share of the observed urban-rural differences. Second, we explicitly quantified the digital environment as a cross-national correlate of linear growth disparities, extending earlier work that often relied on indirect proxies such as media exposure [44]. Although male biological vulnerability is frequently discussed, formal interaction tests did not show statistically significant sex differences in the associations between household environments and HAZ, implying that the socioeconomic and digital constraints identified here are broadly shared across boys and girls in these settings.

The decomposition results indicate that socioeconomic conditions are the dominant correlates of the urban-rural HAZ gap, supporting an interpretation centred on resource inequality rather than rural residence per se [8]. Households with greater economic and educational resources are more likely to secure stable food access [11,15–17,45], demonstrate higher health literacy [46,47], and mobilise care when needed [19,20,44], all of which plausibly shape growth trajectories. Some lower-order contrasts showed negative contributions, likely reflecting compositional complexity in the pooled data rather than contradictory mechanisms. The core message is therefore not that every indicator operates uniformly, but that the aggregate socioeconomic gradient remains the most salient pattern associated with the urban-rural HAZ difference. Importantly, the digital environment remained a substantial secondary component, suggesting that connectivity-related inequalities may add to conventional socioeconomic gaps.

The digital contribution should be interpreted as a conditional, distributional association. In LDCs, device ownership and maternal Internet use likely proxy broader household capacity and may co-vary with unmeasured community factors; residual confounding cannot be fully excluded. Nevertheless, the persistence of a sizeable digital contribution after adjustment for parental education and wealth suggests that these indicators capture dimensions of opportunity not fully represented by conventional measures [48]. Digital access may signal household connectivity – potentially enabling families to bypass geographic barriers, access authoritative health guidance, and participate in financial inclusion [26–28]. On this basis, digital inclusion remains a plausible equity-oriented policy hypothesis that warrants testing with longitudinal or quasi-experimental designs [49].

The physical environment explained 20.3% of the pooled urban-rural HAZ gap, indicating a meaningful but secondary role compared with the socioeconomic and digital domains. Sanitation was the most stable contributor, consistent with a pathway through sustained differences in household hygiene context and pathogen exposure [50,51], which are linked to growth faltering [52]. Other physical indicators (notably water source, handwashing facility, and electricity) showed weaker or less consistent net contributions after simultaneous adjustment, likely reflecting overlap in shared deprivation gradients and the limited granularity of binary classifications.

Our study has several limitations. First, this pooled cross-sectional analysis supports only associative inference. The Blinder-Oaxaca framework decomposes gaps but does not establish causal mechanisms. Second, partial circularity is possible because national urban-rural classifications may incorporate infrastructure-related criteria (*e.g.* electricity and piped water). We therefore interpret physical-environment estimates as descriptive decomposition components and confirmed the robustness of our findings in sensitivity analyses that excluded variables most likely to overlap with residence definitions. Third, the results are sensitive to variable operationalisation. To reduce collinearity with physical-environment indicators, we constructed a harmonised wealth index using durable assets, housing quality, land ownership, and livestock, and interpreted it as a within-country relative socioeconomic ranking. As an additional robustness check, we repeated analyses using the DHS wealth quintiles and obtained similar conclusions. Finally, pooled surveys span 2015–2024 across seven countries, and differences in survey timing may affect comparability, particularly for digital indicators. The pooled estimates should therefore be read as average conditional associations, complemented by country-specific results to show heterogeneity.

We have several policy recommendations. First, strengthening core socioeconomic supports for rural families – poverty reduction, social protection coverage, and parental (especially maternal) education – remains fundamental. Second, digital inclusion may complement nutrition and primary-care programmes through improved connectivity, device access, and caregiver digital health literacy, but its role should be evaluated as a potential lever rather than assumed causal. Third, continued investment in basic infrastructure remains essential, with priority to safe sanitation where associations were most consistent. Finally, implementation should be country-adapted and equity-focused, with routine monitoring of rural subgroups and prospective evaluation of intervention packages to identify which approaches most effectively narrow growth inequalities.

## CONCLUSIONS

The urban-rural disparity in child linear growth across these seven LDCs primarily reflects the unequal distribution of resources within the socioeconomic, digital, and physical environments. This finding suggests that policies aiming to close these multi-sectoral resource gaps may be essential for reducing disparities.

## Additional material


Online Supplementary Document
