# Supplementary material for: Urban-rural disparities in child linear growth: a decomposition analysis of digital, physical, and socioeconomic environments in seven least-developed countries
Source: J Glob Health. 2026 Jul 1;16:04158. doi: 10.7189/jogh.16.04158 (PMC13320656; doi:10.7189/jogh.16.04158)
Supplement: Online Supplementary Document [file jogh-16-04158-s001.pdf]

Supplement to: Liu Z, Wang R, Zhang X, Wu Q. Urban-rural disparities in child linear growth: a decomposition analysis of digital, physical, and socioeconomic environments in seven least developed countries. J Glob Health. 2026;16:04158.

Contents

Supplementary Appendix 1. Descriptive meta-analysis of urban-rural differences in under-five stunting prevalence in UN-classified least developed countries, 2009–2023 2

Supplementary Table S1. Adherence to JoGH GRABDROP reporting items 4

Supplementary Table S2. Tests for effect modification by child sex in associations between household environments and child height-for-age Z-score (HAZ) 8

Supplementary Table S3. Country-specific Blinder-Oaxaca decomposition of the urban-rural gap in child height-for-age Z-score (HAZ) across seven least developed countries 9

Supplementary Table S4. Sensitivity analysis using Fairlie decomposition for the urban-rural gap in child stunting (HAZ < −2 SD) 11

Supplementary Table S5. Sensitivity analysis using original DHS sample weights: Blinder-Oaxaca decomposition of the urban-rural HAZ gap 12

Supplementary Table S6. Complete-case sensitivity analysis: Blinder-Oaxaca decomposition of the urban-rural HAZ gap 13

Supplementary Table S7. Sensitivity analysis using standard DHS wealth quintiles: Blinder-Oaxaca decomposition of the urban-rural HAZ gap 14

Supplementary Table S8. Sensitivity analysis excluding variables highly correlated with urban-rural residence: Blinder-Oaxaca decomposition of the urban-rural HAZ gap 15

Supplementary Table S9. Sensitivity analysis additionally adjusting for immediate determinants: Blinder-Oaxaca decomposition of the urban-rural HAZ gap 16

STROBE Statement—Checklist of items that should be included in reports of *cross-sectional studies* 17

Supplementary Appendix 1. Descriptive meta-analysis of urban-rural differences in under-five stunting prevalence in UN-classified least developed countries, 2009–2023

S1.1 Objective

To provide descriptive context for the magnitude of urban-rural disparities in child linear growth, we summarised the most recent UNICEF-reported prevalence of stunting among children aged 0–59 months, stratified by urban-rural residence, across UN-classified least developed countries (LDCs).

S1.2 Data source and extraction

We used the UNICEF global data platform as the sole data source and extracted country-level prevalence estimates for stunting among children aged 0–59 months, reported separately for urban and rural residence. For each UN-classified LDC, we recorded the most recent observation available during 2009–2023, together with the survey year and source type where reported, such as DHS, MICS, SMART, LSMS, or other nationally representative systems. Data extraction was conducted using a standardised template to ensure consistent capture of residence-specific prevalence estimates and accompanying uncertainty intervals, where available.

S1.3 Eligibility and selection

Eligible records were country-level urban and rural estimates of under-five stunting prevalence from UN-classified LDCs during the prespecified period of 2009–2023. When multiple survey years were available for a country, we retained the most recent estimate to avoid double counting and to reflect the latest reported national situation. Estimates without clear urban-rural stratification were excluded. Because this synthesis used published country-level indicators rather than individual-participant data, no additional harmonisation of sampling frames was possible beyond applying consistent inclusion rules.

S1.4 Outcome definition

The outcome was under-five stunting prevalence, defined as the proportion of children aged 0–59 months with height-for-age z-score (HAZ) below −2 SD, consistent with the World Health Organization Child Growth Standards and UNICEF reporting conventions. Prevalence values were used as reported by UNICEF; we did not re-estimate HAZ distributions or recalculate stunting prevalence from raw survey microdata. Urban and rural categories were also accepted as reported, acknowledging that residence definitions follow country-specific administrative and statistical standards.

S1.5 Statistical synthesis

We pooled rural and urban stunting prevalence separately using inverse-variance-weighted random-effects meta-analysis. For each country-specific estimate, the standard error was derived from the reported 95% confidence interval where available. Forest plots for rural and urban prevalence are presented in Supplementary Figures S1 and S2, respectively.

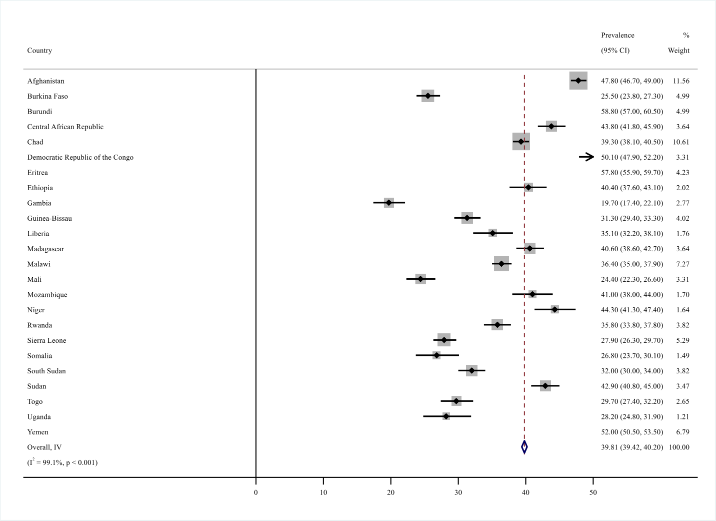

Supplementary Figure S1: Forest plot of rural prevalence (under-5) estimates by country.

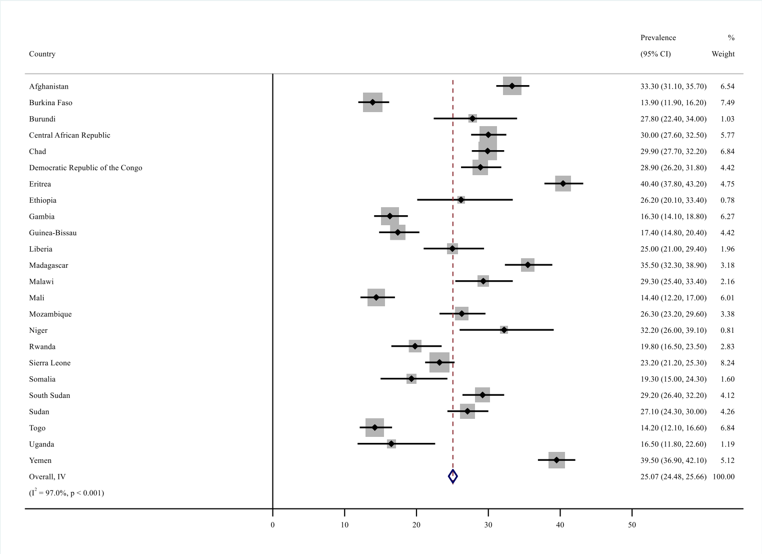

Supplementary Figure S2: Forest plot of urban prevalence (under-5) estimates by country.

S1.6 Data quality, comparability, and interpretive scope

UNICEF compiles estimates from nationally recognised survey programmes and applies standard child growth definitions, supporting broad cross-country comparability at the indicator level. However, differences in survey timing, implementation modality, and country-specific urban-rural classification may introduce heterogeneity. This synthesis used one most recent observation per country and therefore does not capture within-country time trends. Accordingly, the pooled estimates should be interpreted as descriptive background context for the magnitude of residence-based disparities across LDCs, rather than as a formal trend analysis.

Supplementary Table S1. Adherence to JoGH GRABDROP reporting items

**JoGH guideline items**
1. Please list all papers published by each co-author in previous 3 years that were based on secondary analysis of a big data repository
**1)** **Wang R**, Sun Y, Wang H, Yu X, Ma J, Liu Z, Li J, Zou Z, Huang Y. Progress on HIV and other sexually transmitted infections elimination among youth and adults across BRICS-plus countries: Results from the Global Burden of Disease Study 2021. J Infect Public Health. 2025 Feb;18(2):102625. doi: 10.1016/j.jiph.2024.102625. Epub 2024 Dec 20. PMID: 39732057.<br>**2)** Lai Y, Li Y, Liu X, Shi Y, Qu F, **Zhang X**, Shi B, Wang X, Sun T, Huang X, You D. The impact of social and commercial determinants on the unequal increase of oral disorder disease burdens across global, regional, and national contexts. BMC Oral Health. 2025 Aug 9;25(1):1308.<br>**3)** Liu B, **Zhang X**, Lai Y, Sun T, Wang C, Zhao T, Zhang S, Shi B, Li Y, Cui F. Global vaccine confidence trends among adults above and below age 65. npj Vaccines. 2025 Jul 21;10(1):160.<br>**4)** Xu Q, **Zhang X**, Zhao T, Cai X, Zhang S, Wang M, Lu QB, Cui F. Analysis of the trend of notifiable sexually transmitted infections in China between 2006–22. Journal of Global Health. 2025 Jun 27;15:04175.<br>**5)** Lai Y, Chen R, Liu X, Tian W, Wu B, Zhang Y, **Zhang X**, Yang H, Yin F, Xu Y, Miao W. Health poverty reduction effect of medical insurance in China's middle-aged and elderly populations: a cross-sectional study. BMJ open. 2025 Apr 1;15(4):e085226.<br>**6)** Liu X, Wu B, Lai Y, **Zhang X**, Li H, Qu F, Zhang C, Tian Y, Fu X, Li J, Yang H. Temporal Trends in the Burden of Diabetes and Its Risk Factors Across the Western Pacific Region Between 1990 and 2044: A Systematic Analysis of the Global Burden of Disease Study 2019. Diabetes/Metabolism Research and Reviews. 2025 Feb;41(2):e70036.<br>**7)** Wu B, Li Y, Shi B, **Zhang X**, Lai Y, Cui F, Bai X, Xiang W, Geng G, Liu B, Jiao M. Temporal trends of breast cancer burden in the Western Pacific Region from 1990 to 2044: Implications from the Global Burden of Disease Study 2019. Journal of Advanced Research. 2024 May 1;59:189-99.<br>**8)** LAI Yongqiang, LI Ye, **WU Qunhong**, TIAN Yulu, LIU Xinwei, **ZHANG Xiyu**, ZHANG Chenxi, LI Hongyu. Spatial and temporal prevalence of household-based multidimensional health poverty among middle-aged and elderly populations in China: a CHARLS data analysis[J]. Chinese Journal of Public Health, 2023, 39(6): 707-712. (in Chinese)<br>**9)** **ZHANG Xiyu**, LAI Yongqiang, LI Ye, **WU Qunhong**, WU Bing, MIAO Wenqing, ZHANG Chenxi, LIU Xinwei. Spatiotemporal characteristics of health poverty and its associates among middle-aged and elderly populations in China: a CHARLS and statistical data analysis[J]. Chinese Journal of Public Health, 2023, 39(6): 713-719. DOI: 10.11847/zgggws1141548. (in Chinese)<br>**10)** **Zhang X**, Lai Y, Bai X, Wu B, Xiang W, Zhang C, Geng G, Miao W, Xia Q, **Wu Q**, Yang H. Determining the spatial non-stationarity underlying social and natural environment in thyroid cancer in China. Science of The Total Environment. 2023 Apr 20;870:162009.<br>**11)** Wu B, Li Y, Shi B, Zhang X, Lai Y, Cui F, Bai X, Xiang W, Geng G, Liu B, Jiao M, **Wu Q**, Yang H, Zhang C, Liu X, Tian Y, Li H. Temporal trends of breast cancer burden in the Western Pacific Region from 1990 to 2044: Implications from the Global Burden of Disease Study 2019. J Adv Res. 2024 May;59:189-199. doi: 10.1016/j.jare.2023.07.003. Epub 2023 Jul 6. PMID: 37422280; PMCID: PMC11082062.<br>**12)** Liu J, Liu W, Wang L, Wang N, Wu L, Liu X, Liu Z, Zhou Y, Yin X, Liu Y, **Wu Q**, Cui Y, Liang L. Association of Visceral Adiposity Index and Handgrip Strength with Cardiometabolic Multimorbidity among Middle-Aged and Older Adults: Findings from Charls 2011-2020. Nutrients. 2024 Jul 15;16(14):2277. doi: 10.3390/nu16142277. PMID: 39064720; PMCID: PMC11280108.<br>**13)** Liu J, Qi X, Wang R, Zhang J, Lu S, Xie G, Qin Y, Ye D, **Wu Q**. The Physical Developmental Characterization of Children with Nutritional Deficiencies and Attributed Specific Categories. Nutrients. 2024 Dec 28;17(1):86. doi: 10.3390/nu17010086. PMID: 39796520; PMCID: PMC11723147.

14) Liu J, Zhou Y, Guan J, Liu Y, Song W, Liu W, Yin X, Liu Y, Li T, Jin L, Zhang L, Li Y, Wu L, Wang N, Liu Z, Liu X, Wang Y, **Wu Q**, Liang L. Global burden of tuberculosis among adults aged 60 years and older, 1990-2021: Findings from the global burden of disease study 2021. Int J Infect Dis. 2025 Sep;158:107966. doi: 10.1016/j.ijid.2025.107966. Epub 2025 Jun 26. PMID: 40581250.

15) Guo P, Wang R, Li J, Qin Y, Meng N, Shan L, Liu H, Liu J, **Wu Q**. Temporal and spatial convergence: the major depressive disorder burden attributed to intimate partner violence against women. Eur J Psychotraumatol. 2024;15(1):2386226. doi: 10.1080/20008066.2024.2386226. Epub 2024 Oct 2. PMID: 39355978; PMCID: PMC11448340.

16) Wang Q, Wei M, Meng N, Wang P, Wang Y, Liu H, Zhang X, Zhuge R, Wang Y, Yu T, Gao Y, Wang J, Wang K, **Wu Q**. Associations between edentulism and risk of neurodegenerative diseases among middle-aged and older adults in China: a decade-long cohort study. Sci Rep. 2026 Jan 13;16(1):3931. doi: 10.1038/s41598-025-34017-z. PMID: 41526419; PMCID: PMC12855896.

17) Liu J, Liu Z, Liu X, Wang N, Wu L, Xu X, Liu W, Feng Y, Zhang H, Zhou Y, Yin X, Liu Y, **Wu Q**, Ning N, Liang L. Combined exposure to mixed brominated flame retardants on obstructive sleep apnea syndrome in US adults. BMC Public Health. 2025 Jan 14;25(1):146. doi: 10.1186/s12889-024-21204-2. PMID: 39806355; PMCID: PMC11730165.

18) Huang W, Yin L, Li H, Yang W, Huang S, Wang L, Wang K, Hao Y, **Wu Q**, Liu H. Impact of temperature variations on burden of lower respiratory infections under climate change (1990-2021). BMC Public Health. 2025 May 28;25(1):1972. doi: 10.1186/s12889-025-23203-3. PMID: 40437426; PMCID: PMC12117812.

19) Liu J, Liu C, Cui Y, Liu Z, Feng Y, Wang Y, Guan L, Liu W, Zhang H, Liu X, Wu L, Liu Z, Wang N, Yang A, **Wu Q**, Liang L. Social participation and exposure to tuberculosis education: a cross-sectional study of older (≥ 60 years) domestic migrants in China. BMC Public Health. 2025 Feb 17;25(1):646. doi: 10.1186/s12889-025-21779-4. PMID: 39962428; PMCID: PMC11834676.

20) Lai Y, Chen R, Liu X, Tian W, Wu B, Zhang Y, Zhang X, Yang H, Yin F, Xu Y, Miao W, Shan L, **Wu Q**, Shi B, Leng F, Li Y. Health poverty reduction effect of medical insurance in China's middle-aged and elderly populations: a cross-sectional study. BMJ Open. 2025 Apr 27;15(4):e085226. doi: 10.1136/bmjopen-2024-085226. PMID: 40288789; PMCID: PMC12035475.

21) Liu J, Yin H, Wang N, Wang Y, Guan L, Feng Y, Wu L, Liu W, Zhang H, Liu Z, Liu X, Zhou Y, Liu Y, Yin X, **Wu Q**, Jiao M, Liu C, Liang L. Factors associated with exposure to tuberculosis education among internal migrants with diabetes in China: a multilevel regression analysis of cross-sectional data from the 2017 China Migrants Dynamic Survey. BMJ Open. 2025 Apr 8;15(4):e086915. doi: 10.1136/bmjopen-2024-086915. PMID: 40204299; PMCID: PMC11979509.

2. Please explain the key elements of your study design and the use of the available datasets that make your study an original scientific contribution

Our study used a pooled, multi-country, cross-sectional design based on secondary analysis of Demographic and Health Survey (DHS) data from seven UN-classified least developed countries (LDCs). These countries were selected because they had recent DHS data, available digital-environment indicators, and acceptable anthropometric data completeness. The DHS uses a two-stage stratified cluster sampling design, with enumeration areas selected first and households selected second, enabling nationally representative estimates within each country.

The study's originality lies in both measurement and analytic strategy. First, we constructed a multidimensional household-environment framework that explicitly included a digital domain, measured through household device access, maternal internet use, and electronic financial accounts, alongside physical and socioeconomic domains. This approach allowed us to examine digital disparities as a distinct component of household resources in relation to urban-rural differences in child linear growth. Second, rather than reporting only adjusted regression coefficients, we used Blinder-Oaxaca decomposition to partition the predicted urban-rural HAZ gap into explained and unexplained components and to quantify the relative contributions of socioeconomic, digital, and physical household environments.

3. Please list all publications that addressed similar research questions in the same dataset and indicate where you cited them in your paper

1) Sharaf MF, Rashad AS. Regional inequalities in child malnutrition in Egypt, Jordan, and Yemen: a Blinder-Oaxaca decomposition analysis. Health Econ Rev. 2016 Dec;6(1):23. doi: 10.1186/s13561-016-0097-3. Epub 2016 Jun 7. PMID: 27271178; PMCID: PMC4894857.

2) Tadesse SE, Mekonnen TC, Dewau R, Zerga AA, Kebede N, Feleke YW, Muche A. Urban-rural disparity in stunting among Ethiopian children aged 6-59 months old: A multivariate decomposition analysis of 2019 Mini-EDHS. PLoS One. 2023 Apr 19;18(4):e0284382. doi: 10.1371/journal.pone.0284382. PMID: 37075042; PMCID: PMC10115250.

3) Sriram S, Naz L. Inequality of opportunity in child nutrition in Pakistan. PLoS One. 2025 Feb 21;20(2):e0318425. doi: 10.1371/journal.pone.0318425. PMID: 39982904; PMCID: PMC11844883.

To our knowledge, no previous study has used the same pooled seven-country LDC DHS dataset to examine the contribution of socioeconomic, digital, and physical household environments to urban-rural differences in child HAZ. We identified several studies that used DHS or similar nationally representative datasets and decomposition methods to examine inequalities in child nutrition, including studies from Egypt, Jordan, and Yemen; Ethiopia; and Pakistan. These studies are cited in the Introduction and Discussion as relevant methodological and substantive comparators.

4. Please explain how you addressed multiple testing through an appropriately rigorous statistical threshold and indicate this in the methods section

We addressed multiplicity by defining the primary interpretation at the domain level rather than treating all individual decomposition terms as independent primary hypotheses. For effect modification by child sex, we used joint Wald tests for each environmental domain, evaluating whether the set of interaction terms between child sex and domain-specific indicators was jointly different from zero. This approach reduced reliance on multiple isolated interaction tests.

For the Blinder-Oaxaca decomposition, statistical interpretation focused primarily on the aggregate digital, physical, and socioeconomic domains. Individual indicator-level contributions were interpreted descriptively to characterise the composition of each domain rather than as separate confirmatory hypothesis tests. We also assessed the stability of findings across six prespecified sensitivity analyses, including complete-case analysis, alternative weighting, and non-linear decomposition. Statistical significance was assessed using two-sided P values < 0.05.

5. Please declare to what extent have AI chatbots been used in developing your paper and to which parts of the paper did they contribute
After drafting the manuscript, the authors used ChatGPT only to improve language clarity and readability. The tool was not used to generate data, perform statistical analyses, create tables or figures, or draw scientific conclusions. After using the tool, the authors reviewed and edited all content as needed and take full responsibility for the final manuscript.

Supplementary Table S2. Tests for effect modification by child sex in associations between household environments and child height-for-age Z-score (HAZ)

Interaction Terms	Test method	P
Child sex × Residence	Standard Wald test	0.864
Child sex × Household Environment Domains		
Child sex × Digital environment	Joint Wald test	0.788
Child sex × Physical environment	Joint Wald test	0.392
Child sex × Socioeconomic environment	Joint Wald test	0.072

Supplementary Table S3. Country-specific Blinder-Oaxaca decomposition of the urban-rural gap in child height-for-age Z-score (HAZ) across seven least developed countries

Factor	Benin (2017-18)	Ethiopia (2016)	Haiti (2016-17)	Mali (2023-24)	Myanmar (2015-16)	Timor-Leste (2016)	Zambia (2018-19)
	β (95% CI)	β (95% CI)	β (95% CI)	β (95% CI)	β (95% CI)	β (95% CI)	β (95% CI)
**Digital environment**	**0.040 (0.023, 0.057)**	**0.058 (-0.027, 0.142)**	**0.099 (0.051, 0.147)**	**0.104 (0.068, 0.139)**	**0.086 (0.008, 0.163)**	**0.030 (-0.042, 0.103)**	**0.143 (0.082, 0.204)**
Household digital access (yes vs. no)	0.007 (0.000, 0.013)	0.089 (0.037, 0.141)	0.047 (0.022, 0.072)	-0.001 (-0.003, 0.002)	0.045 (0.008, 0.082)	0.007 (-0.017, 0.032)	0.027 (-0.003, 0.057)
Maternal internet use (weekly vs. never)	0.004 (-0.001, 0.008)	0.018 (-0.001, 0.037)	0.010 (-0.002, 0.022)	0.027 (0.016, 0.038)	0.005 (-0.017, 0.028)	0.009 (-0.015, 0.033)	0.008 (-0.006, 0.023)
Maternal internet use (daily vs. never)	0.013 (0.005, 0.021)	0.019 (0.004, 0.034)	0.022 (0.002, 0.043)	0.067 (0.043, 0.091)	0.031 (-0.026, 0.088)	0.049 (0.005, 0.092)	0.049 (0.024, 0.074)
Electronic financial account (yes vs. no)	0.017 (0.004, 0.029)	-0.068 (-0.150, 0.014)	0.019 (-0.012, 0.050)	0.011 (-0.010, 0.031)	0.004 (-0.028, 0.036)	-0.034 (-0.077, 0.009)	0.059 (0.018, 0.100)
**Physical environment**	**0.027 (-0.001, 0.056)**	**0.106 (-0.084, 0.296)**	**0.061 (-0.017, 0.139)**	**0.090 (0.048, 0.133)**	**0.031 (-0.058, 0.119)**	**-0.018 (-0.136, 0.101)**	**0.091 (-0.031, 0.214)**
Cooking fuel (clean vs. solid)	0.011 (0.002, 0.021)	0.012 (-0.073, 0.097)	0.026 (0.008, 0.044)	-0.001 (-0.005, 0.002)	0.009 (-0.061, 0.079)	-0.022 (-0.061, 0.018)	0.042 (0.008, 0.077)
Drinking water (improved vs. unimproved)	0.007 (-0.002, 0.015)	-0.035 (-0.093, 0.023)	-0.002 (-0.013, 0.008)	0.001 (-0.012, 0.014)	0.005 (-0.016, 0.027)	0.009 (-0.044, 0.061)	0.015 (-0.017, 0.047)
Sanitation facility (improved vs. unimproved)	0.011 (-0.009, 0.032)	0.113 (0.038, 0.189)	0.057 (0.015, 0.100)	0.005 (-0.011, 0.021)	0.003 (-0.025, 0.032)	0.037 (-0.027, 0.100)	-0.020 (-0.059, 0.020)
Handwashing (present vs. absent)	-0.000 (-0.002, 0.001)	-0.019 (-0.054, 0.016)	0.007 (-0.004, 0.017)	0.001 (-0.003, 0.005)	0.016 (-0.002, 0.034)	0.011 (-0.005, 0.028)	0.005 (-0.012, 0.022)
Electricity availability (yes vs. no)	-0.001 (-0.025, 0.022)	0.002 (-0.162, 0.165)	-0.018 (-0.089, 0.053)	0.087 (0.046, 0.128)	-0.010 (-0.070, 0.049)	-0.057 (-0.134, 0.019)	0.049 (-0.057, 0.156)
Housing density (not overcrowded vs. overcrowded)	-0.001 (-0.003, 0.002)	0.033 (0.007, 0.059)	-0.008 (-0.016, 0.000)	-0.003 (-0.007, 0.001)	0.007 (-0.004, 0.018)	0.005 (-0.004, 0.013)	-0.000 (-0.003, 0.002)
**Socioeconomic environment**	**0.104 (0.062, 0.146)**	**0.355 (0.182, 0.528)**	**0.315 (0.190, 0.440)**	**0.114 (0.065, 0.162)**	**0.133 (0.050, 0.217)**	**0.178 (0.047, 0.309)**	**0.069 (-0.046, 0.183)**
Household wealth (poorer vs. poorest)	-0.014 (-0.029, 0.001)	-0.048 (-0.095, -0.001)	-0.013 (-0.049, 0.023)	-0.023 (-0.042, -0.003)	0.000 (-0.020, 0.021)	0.001 (-0.034, 0.035)	0.002 (-0.033, 0.037)
Household wealth (middle vs. poorest)	-0.022 (-0.037, -0.007)	-0.016 (-0.032, -0.000)	-0.020 (-0.035, -0.005)	-0.009 (-0.017, -0.001)	0.000 (-0.011, 0.011)	0.000 (-0.033, 0.033)	-0.002 (-0.007, 0.003)
Household wealth (richer vs. poorest)	0.025 (0.010, 0.040)	-0.009 (-0.024, 0.006)	0.127 (0.052, 0.201)	0.011 (0.001, 0.021)	0.008 (-0.011, 0.027)	-0.000 (-0.017, 0.016)	0.024 (-0.029, 0.076)
Household wealth (richest vs. poorest)	0.090 (0.053, 0.127)	0.243 (0.076, 0.411)	0.146 (0.073, 0.219)	0.078 (0.030, 0.125)	0.062 (-0.007, 0.132)	0.205 (0.060, 0.351)	0.025 (-0.061, 0.111)
Maternal education (primary vs. none)	-0.000 (-0.003, 0.003)	0.001 (-0.007, 0.010)	-0.008 (-0.028, 0.013)	-0.001 (-0.002, 0.001)	-0.021 (-0.068, 0.027)	0.011 (-0.018, 0.039)	-0.012 (-0.055, 0.032)
Maternal education (secondary+ vs. none)	0.015 (0.003, 0.026)	0.119 (0.029, 0.209)	0.036 (-0.027, 0.098)	0.025 (0.002, 0.049)	0.088 (0.005, 0.171)	-0.005 (-0.075, 0.066)	0.049 (-0.012, 0.110)
Paternal education (primary vs. none)	0.000 (-0.002, 0.003)	-0.012 (-0.029, 0.005)	-0.013 (-0.039, 0.013)	0.003 (-0.001, 0.006)	-0.017 (-0.051, 0.017)	-0.003 (-0.031, 0.025)	-0.001 (-0.058, 0.056)
Paternal education (secondary+ vs. none)	0.010 (-0.003, 0.024)	0.076 (-0.013, 0.165)	0.060 (0.003, 0.117)	0.028 (0.004, 0.053)	0.012 (-0.052, 0.075)	-0.031 (-0.096, 0.035)	-0.016 (-0.086, 0.053)
**Covariates**	**-0.007 (-0.020, 0.006)**	**-0.007 (-0.068, 0.053)**	**0.002 (-0.027, 0.030)**	**-0.006 (-0.020, 0.008)**	**0.041 (-0.010, 0.092)**	**-0.026 (-0.064, 0.011)**	**-0.024 (-0.046, -0.002)**
Child sex (female vs. male)	0.002 (-0.002, 0.006)	-0.003 (-0.010, 0.005)	-0.001 (-0.006, 0.004)	0.002 (-0.001, 0.004)	0.002 (-0.005, 0.008)	-0.004 (-0.013, 0.005)	0.001 (-0.006, 0.007)
Child age (12-23 mo vs. 0-11 mo)	0.008 (-0.001, 0.016)	-0.013 (-0.055, 0.029)	0.002 (-0.012, 0.015)	-0.001 (-0.012, 0.010)	-0.025 (-0.052, 0.003)	0.013 (-0.011, 0.037)	-0.001 (-0.016, 0.014)
Child age (24-35 mo vs. 0-11 mo)	-0.015 (-0.026, -0.003)	0.026 (-0.018, 0.071)	0.010 (-0.013, 0.033)	0.003 (-0.009, 0.015)	-0.017 (-0.056, 0.022)	-0.006 (-0.031, 0.020)	0.002 (-0.013, 0.017)
Child age (36-47 mo vs. 0-11 mo)	0.004 (-0.007, 0.014)	0.024 (-0.018, 0.067)	-0.001 (-0.017, 0.015)	0.001 (-0.009, 0.012)	0.006 (-0.034, 0.045)	-0.004 (-0.042, 0.033)	-0.023 (-0.038, -0.007)

Child age (48-59 mo vs. 0-11 mo)	-0.001 (-0.010, 0.007)	0.023 (-0.020, 0.065)	-0.015 (-0.028, -0.002)	-0.000 (-0.005, 0.005)	0.063 (0.028, 0.098)	-0.017 (-0.044, 0.010)	0.006 (-0.002, 0.015)
Birth order (2-4 vs. 1st)	0.007 (0.003, 0.012)	0.016 (-0.001, 0.033)	0.004 (-0.001, 0.009)	0.006 (0.001, 0.011)	0.004 (-0.003, 0.010)	0.001 (-0.003, 0.005)	0.007 (-0.001, 0.015)
Birth order (≥5 vs. 1st)	-0.012 (-0.019, -0.005)	-0.061 (-0.105, -0.016)	0.001 (-0.018, 0.020)	-0.016 (-0.025, -0.007)	0.013 (-0.008, 0.033)	-0.008 (-0.021, 0.004)	-0.017 (-0.033, -0.000)
Sex of household head (female vs. male)	0.000 (-0.001, 0.002)	-0.021 (-0.040, -0.001)	0.002 (-0.002, 0.007)	-0.000 (-0.005, 0.004)	-0.004 (-0.011, 0.003)	-0.000 (-0.003, 0.003)	0.000 (-0.002, 0.002)
**Total explained gap**	**0.165 (0.121, 0.209)**	**0.511 (0.296, 0.726)**	**0.476 (0.363, 0.590)**	**0.302 (0.240, 0.363)**	**0.291 (0.183, 0.399)**	**0.164 (0.016, 0.313)**	**0.279 (0.176, 0.383)**
**Total unexplained gap**	**0.083 (0.008, 0.158)**	**-0.090 (-0.353, 0.173)**	**-0.291 (-0.423, -0.159)**	**0.125 (0.036, 0.213)**	**0.154 (-0.003, 0.310)**	**-0.110 (-0.306, 0.086)**	**-0.080 (-0.209, 0.048)**
**Total predicted gap**	**0.248 (0.176, 0.319)**	**0.421 (0.223, 0.619)**	**0.185 (0.067, 0.303)**	**0.426 (0.338, 0.514)**	**0.445 (0.303, 0.586)**	**0.054 (-0.136, 0.244)**	**0.199 (0.102, 0.295)**

Supplementary Table S4. Sensitivity analysis using Fairlie decomposition for the urban-rural gap in child stunting (HAZ < −2 SD)

Factor	β	95% CI		P-value
		Lower	Upper	
**Digital environment**	**-0.022**	**-0.033**	**-0.011**	**<0.001**
Household digital access (yes vs. no)	-0.011	-0.018	-0.004	0.003
Maternal internet use (weekly vs. never)	-0.004	-0.006	-0.003	<0.001
Maternal internet use (daily vs. never)	-0.009	-0.011	-0.006	<0.001
Electronic financial account (yes vs. no)	0.002	-0.006	0.010	0.631
**Physical environment**	**-0.034**	**-0.050**	**-0.017**	**<0.001**
Cooking fuel (clean vs. solid)	-0.005	-0.011	0.002	0.188
Drinking water (improved vs. unimproved)	-0.002	-0.007	0.004	0.523
Sanitation facility (improved vs. unimproved)	-0.006	-0.014	0.002	0.124
Handwashing (present vs. absent)	0.000	-0.003	0.003	0.993
Electricity availability (yes vs. no)	-0.014	-0.028	-0.001	0.035
Housing density (not overcrowded vs. overcrowded)	-0.007	-0.009	-0.004	<0.001
**Socioeconomic environment**	**-0.066**	**-0.084**	**-0.048**	**<0.001**
Household wealth (poorer vs. poorest)	0.005	-0.001	0.011	0.085
Household wealth (middle vs. poorest)	0.001	0.000	0.001	0.002
Household wealth (richer vs. poorest)	-0.006	-0.009	-0.003	<0.001
Household wealth (richest vs. poorest)	-0.035	-0.049	-0.021	<0.001
Maternal education (primary vs. none)	0.000	0.000	0.001	0.192
Maternal education (secondary+ vs. none)	-0.021	-0.031	-0.010	<0.001
Paternal education (primary vs. none)	0.005	0.001	0.008	0.008
Paternal education (secondary+ vs. none)	-0.015	-0.026	-0.004	0.006
**Covariates**	**0.004**	**-0.001**	**0.009**	**0.085**
Child sex (female vs. male)	0.000	-0.001	<0.001	<0.001
Child age (12-23 mo vs. 0-11 mo)	-0.002	-0.003	-0.001	<0.001
Child age (24-35 mo vs. 0-11 mo)	0.000	0.000	0.001	0.454
Child age (36-47 mo vs. 0-11 mo)	0.001	0.001	0.002	<0.001
Child age (48-59 mo vs. 0-11 mo)	0.002	0.001	0.002	0.001
Birth order (2-4 vs. 1st)	-0.002	-0.003	0.000	0.032
Birth order (≥5 vs. 1st)	0.004	-0.001	0.009	0.112
Sex of household head (female vs. male)	0.001	-0.001	0.003	0.236
**Country fixed effects**	**-0.002**	**-0.008**	**0.004**	**0.517**

Supplementary Table S5. Sensitivity analysis using original DHS sample weights: Blinder-Oaxaca decomposition of the urban-rural HAZ gap

Factor	β	95% CI		P-value	Percentage explained
		Lower	Upper		(PE, %)
**Digital environment**	**0.081**	**0.063**	**0.100**	**<0.001**	**32.0**
Household digital access (yes vs. no)	0.029	0.019	0.038	<0.001	11.5
Maternal internet use (weekly vs. never)	0.011	0.007	0.016	<0.001	4.3
Maternal internet use (daily vs. never)	0.037	0.027	0.046	<0.001	14.6
Electronic financial account (yes vs. no)	0.004	-0.008	0.017	0.475	1.6
**Physical environment**	**0.054**	**0.026**	**0.082**	**<0.001**	**21.3**
Cooking fuel (clean vs. solid)	0.010	0.000	0.020	0.061	4.0
Drinking water (improved vs. unimproved)	0.000	-0.008	0.008	0.980	0.0
Sanitation facility (improved vs. unimproved)	0.017	0.004	0.030	0.010	6.7
Handwashing (present vs. absent)	0.002	-0.001	0.005	0.225	0.8
Electricity availability (yes vs. no)	0.022	-0.002	0.046	0.077	8.7
Housing density (not overcrowded vs. overcrowded)	0.004	0.001	0.006	0.004	1.6
**Socioeconomic environment**	**0.144**	**0.112**	**0.177**	**<0.001**	**56.9**
Household wealth (poorer vs. poorest)	-0.013	-0.024	-0.001	0.032	-5.1
Household wealth (middle vs. poorest)	-0.008	-0.013	-0.004	<0.001	-3.2
Household wealth (richer vs. poorest)	0.018	0.009	0.027	<0.001	7.1
Household wealth (richest vs. poorest)	0.099	0.071	0.128	<0.001	39.1
Maternal education (primary vs. none)	-0.002	-0.005	0.002	0.339	-0.8
Maternal education (secondary+ vs. none)	0.033	0.017	0.050	<0.001	13.0
Paternal education (primary vs. none)	-0.009	-0.014	-0.003	0.002	-3.6
Paternal education (secondary+ vs. none)	0.025	0.008	0.041	0.003	9.9
**Covariates**	**-0.009**	**-0.019**	**0.000**	**0.058**	**-3.6**
Child sex (female vs. male)	0.001	-0.001	0.003	0.518	0.4
Child age (12-23 mo vs. 0-11 mo)	0.000	-0.006	0.006	0.985	0.0
Child age (24-35 mo vs. 0-11 mo)	-0.001	-0.009	0.006	0.726	-0.4
Child age (36-47 mo vs. 0-11 mo)	-0.002	-0.009	0.006	0.685	-0.8
Child age (48-59 mo vs. 0-11 mo)	0.003	-0.002	0.009	0.207	1.2
Birth order (2-4 vs. 1st)	0.005	0.002	0.007	<0.001	2.0
Birth order (≥5 vs. 1st)	-0.015	-0.022	-0.009	<0.001	-5.9
Sex of household head (female vs. male)	0.000	-0.002	0.002	0.981	0.0
**Country fixed effects**	**-0.017**	**-0.036**	**0.002**	**0.081**	**-6.7**
**Total explained gap**	0.253	0.213	0.294	<0.001	100.0
**Total unexplained gap**	0.007	-0.042	0.057	0.769	-
**Total predicted gap**	0.261	0.211	0.311	<0.001	-

Supplementary Table S6. Complete-case sensitivity analysis: Blinder-Oaxaca decomposition of the urban-rural HAZ gap

Factor	β	95% CI		P-value	Percentage explained
		Lower	Upper		(PE, %)
**Digital environment**	**0.077**	**0.063**	**0.091**	**<0.001**	**30.8**
Household digital access (yes vs. no)	0.030	0.023	0.037	<0.001	12.0
Maternal internet use (weekly vs. never)	0.009	0.006	0.012	<0.001	3.5
Maternal internet use (daily vs. never)	0.027	0.021	0.033	<0.001	10.8
Electronic financial account (yes vs. no)	0.011	0.001	0.022	0.037	4.4
**Physical environment**	**0.052**	**0.031**	**0.072**	**<0.001**	**20.7**
Cooking fuel (clean vs. solid)	0.014	0.007	0.021	<0.001	5.7
Drinking water (improved vs. unimproved)	0.003	-0.002	0.009	0.254	1.3
Sanitation facility (improved vs. unimproved)	0.010	-0.001	0.020	0.084	3.8
Handwashing (present vs. absent)	0.001	-0.001	0.003	0.243	0.5
Electricity availability (yes vs. no)	0.020	0.002	0.037	0.026	7.9
Housing density (not overcrowded vs. overcrowded)	0.003	0.002	0.005	<0.001	1.4
**Socioeconomic environment**	**0.150**	**0.126**	**0.175**	**<0.001**	**60.0**
Household wealth (poorer vs. poorest)	-0.009	-0.017	0.000	0.054	-3.4
Household wealth (middle vs. poorest)	-0.005	-0.008	-0.002	<0.001	-2.1
Household wealth (richer vs. poorest)	0.015	0.009	0.020	<0.001	5.9
Household wealth (richest vs. poorest)	0.104	0.082	0.126	<0.001	41.5
Maternal education (primary vs. none)	-0.001	-0.003	0.001	0.177	-0.5
Maternal education (secondary+ vs. none)	0.030	0.018	0.043	<0.001	12.1
Paternal education (primary vs. none)	-0.004	-0.007	-0.001	0.004	-1.7
Paternal education (secondary+ vs. none)	0.021	0.008	0.033	0.001	8.2
**Covariates**	**0.002**	**-0.006**	**0.010**	**0.669**	**0.7**
Child sex (female vs. male)	0.000	-0.001	0.002	0.551	0.2
Child age (12-23 mo vs. 0-11 mo)	-0.005	-0.012	0.001	0.088	-2.2
Child age (24-35 mo vs. 0-11 mo)	0.002	-0.005	0.010	0.560	0.9
Child age (36-47 mo vs. 0-11 mo)	0.005	-0.002	0.012	0.128	2.1
Child age (48-59 mo vs. 0-11 mo)	0.003	-0.002	0.008	0.221	1.3
Birth order (2-4 vs. 1st)	0.005	0.002	0.008	0.001	1.9
Birth order (≥5 vs. 1st)	-0.010	-0.015	-0.005	<0.001	-4.0
Sex of household head (female vs. male)	0.001	0.000	0.002	0.018	0.5
**Country fixed effects**	**-0.031**	**-0.037**	**-0.024**	**<0.001**	**-12.2**
**Total explained gap**	0.251	0.228	0.273	<0.001	100.0
**Total unexplained gap**	0.010	-0.025	0.045	0.567	-
**Total predicted gap**	0.261	0.231	0.291	<0.001	-

Supplementary Table S7. Sensitivity analysis using standard DHS wealth quintiles: Blinder-Oaxaca decomposition of the urban-rural HAZ gap

Factor	β	95% CI		P-value	Percentage explained
		Lower	Upper		(PE, %)
**Digital environment**	**0.091**	**0.055**	**0.126**	**<0.001**	**25.0**
Household digital access (yes vs. no)	0.059	0.033	0.085	<0.001	16.4
Maternal internet use (weekly vs. never)	0.014	0.008	0.020	<0.001	3.9
Maternal internet use (daily vs. never)	0.034	0.022	0.046	<0.001	9.5
Electronic financial account (yes vs. no)	-0.017	-0.046	0.011	0.238	-4.7
**Physical environment**	**0.051**	**-0.009**	**0.111**	**0.093**	**14.2**
Cooking fuel (clean vs. solid)	0.013	-0.013	0.039	0.328	3.6
Drinking water (improved vs. unimproved)	-0.016	-0.040	0.007	0.168	-4.5
Sanitation facility (improved vs. unimproved)	0.035	0.009	0.060	0.007	9.6
Handwashing (present vs. absent)	-0.003	-0.016	0.009	0.596	-1.0
Electricity availability (yes vs. no)	0.007	-0.041	0.055	0.766	2.0
Housing density (not overcrowded vs. overcrowded)	0.016	0.008	0.025	<0.001	4.5
**Socioeconomic environment**	**0.258**	**0.185**	**0.331**	**<0.001**	**71.2**
Household wealth (poorer vs. poorest)	-0.010	-0.032	0.012	0.380	-2.7
Household wealth (middle vs. poorest)	-0.024	-0.040	-0.008	0.004	-6.6
Household wealth (richer vs. poorest)	0.016	0.005	0.027	0.006	4.3
Household wealth (richest vs. poorest)	0.178	0.105	0.252	<0.001	49.3
Maternal education (primary vs. none)	-0.001	-0.006	0.003	0.535	-0.4
Maternal education (secondary+ vs. none)	0.066	0.031	0.101	<0.001	18.2
Paternal education (primary vs. none)	-0.015	-0.029	-0.002	0.029	-4.2
Paternal education (secondary+ vs. none)	0.049	0.012	0.085	0.009	13.5
**Covariates**	**-0.011**	**-0.034**	**0.011**	**0.328**	**-3.1**
Child sex (female vs. male)	0.000	-0.003	0.003	0.841	0.1
Child age (12-23 mo vs. 0-11 mo)	-0.009	-0.023	0.005	0.193	-2.5
Child age (24-35 mo vs. 0-11 mo)	0.002	-0.013	0.017	0.800	0.6
Child age (36-47 mo vs. 0-11 mo)	0.003	-0.011	0.017	0.666	0.9
Child age (48-59 mo vs. 0-11 mo)	0.020	0.007	0.033	0.003	5.4
Birth order (2-4 vs. 1st)	0.006	0.001	0.011	0.031	1.5
Birth order (≥5 vs. 1st)	-0.027	-0.044	-0.009	0.002	-7.4
Sex of household head (female vs. male)	-0.006	-0.012	0.000	0.044	-1.7
**Country fixed effects**	**-0.027**	**-0.052**	**-0.001**	**0.039**	**-7.4**
**Total explained gap**	0.362	0.292	0.432	<0.001	100.0
**Total unexplained gap**	-0.002	-0.086	0.082	0.969	-
**Total predicted gap**	0.360	0.279	0.441	<0.001	-

Supplementary Table S8. Sensitivity analysis excluding variables highly correlated with urban-rural residence: Blinder-Oaxaca decomposition of the urban-rural HAZ gap

Factor	β	95% CI		P-value	Percentage explained
		Lower	Upper		(PE, %)
**Digital environment**	**0.103**	**0.067**	**0.138**	**<0.001**	**31.7**
Household digital access (yes vs. no)	0.062	0.036	0.088	<0.001	19.1
Maternal internet use (weekly vs. never)	0.015	0.010	0.021	<0.001	4.7
Maternal internet use (daily vs. never)	0.038	0.026	0.050	<0.001	11.7
Electronic financial account (yes vs. no)	-0.012	-0.041	0.016	0.397	-3.8
**Physical environment**	**0.014**	**-0.001**	**0.029**	**0.076**	**4.2**
Handwashing (present vs. absent)	-0.002	-0.015	0.010	0.731	-0.7
Housing density (not overcrowded vs. overcrowded)	0.016	0.008	0.024	<0.001	4.9
**Socioeconomic environment**	**0.244**	**0.182**	**0.306**	**<0.001**	**75.4**
Household wealth (poorer vs. poorest)	-0.025	-0.048	-0.002	0.030	-7.9
Household wealth (middle vs. poorest)	-0.006	-0.011	-0.001	0.013	-1.9
Household wealth (richer vs. poorest)	0.017	0.007	0.028	0.001	5.4
Household wealth (richest vs. poorest)	0.152	0.097	0.207	<0.001	47.0
Maternal education (primary vs. none)	-0.002	-0.006	0.003	0.476	-0.5
Maternal education (secondary+ vs. none)	0.071	0.037	0.105	<0.001	22.0
Paternal education (primary vs. none)	-0.017	-0.031	-0.002	0.024	-5.1
Paternal education (secondary+ vs. none)	0.053	0.016	0.091	0.005	16.5
**Covariates**	**-0.010**	**-0.033**	**0.013**	**0.393**	**-3.1**
Child sex (female vs. male)	0.000	-0.003	0.003	0.841	0.1
Child age (12-23 mo vs. 0-11 mo)	-0.009	-0.023	0.005	0.193	-2.8
Child age (24-35 mo vs. 0-11 mo)	0.002	-0.013	0.017	0.800	0.6
Child age (36-47 mo vs. 0-11 mo)	0.003	-0.011	0.018	0.666	1.0
Child age (48-59 mo vs. 0-11 mo)	0.020	0.007	0.032	0.003	6.1
Birth order (2-4 vs. 1st)	0.006	0.001	0.011	0.031	1.7
Birth order (≥5 vs. 1st)	-0.025	-0.043	-0.008	0.004	-7.9
Sex of household head (female vs. male)	-0.006	-0.012	0.000	0.058	-1.8
**Country fixed effects**	**-0.027**	**-0.053**	**-0.001**	**0.043**	**-8.3**
**Total explained gap**	0.323	0.261	0.386	<0.001	100.0
**Total unexplained gap**	0.037	-0.050	0.124	0.403	-
**Total predicted gap**	0.360	0.279	0.442	<0.001	-

Supplementary Table S9. Sensitivity analysis additionally adjusting for immediate determinants: Blinder-Oaxaca decomposition of the urban-rural HAZ gap

Factor	β	95% CI		P-value	Percentage explained
		Lower	Upper		(PE, %)
**Digital environment**	**0.110**	**0.050**	**0.171**	**<0.001**	**36.1**
Household digital access (yes vs. no)	0.090	0.042	0.138	<0.001	29.5
Maternal internet use (weekly vs. never)	0.012	0.003	0.020	0.009	3.8
Maternal internet use (daily vs. never)	0.029	0.009	0.049	0.005	9.4
Electronic financial account (yes vs. no)	-0.020	-0.067	0.027	0.399	-6.6
**Physical environment**	**0.099**	**0.004**	**0.194**	**0.041**	**32.4**
Cooking fuel (clean vs. solid)	0.020	-0.034	0.073	0.465	6.5
Drinking water (improved vs. unimproved)	-0.016	-0.046	0.015	0.310	-5.2
Sanitation facility (improved vs. unimproved)	0.043	-0.001	0.087	0.054	14.1
Handwashing (present vs. absent)	-0.005	-0.026	0.017	0.668	-1.5
Electricity availability (yes vs. no)	0.039	-0.039	0.116	0.327	12.7
Housing density (not overcrowded vs. overcrowded)	0.018	0.003	0.033	0.017	5.9
**Socioeconomic environment**	**0.165**	**0.066**	**0.264**	**0.001**	**54.1**
Household wealth (poorer vs. poorest)	-0.002	-0.047	0.044	0.945	-0.5
Household wealth (middle vs. poorest)	0.001	-0.004	0.007	0.655	0.4
Household wealth (richer vs. poorest)	0.005	-0.005	0.015	0.324	1.7
Household wealth (richest vs. poorest)	0.055	-0.044	0.154	0.277	18.0
Maternal education (primary vs. none)	0.003	-0.009	0.015	0.625	1.0
Maternal education (secondary+ vs. none)	0.062	0.004	0.120	0.035	20.4
Paternal education (primary vs. none)	-0.023	-0.046	-0.001	0.045	-7.7
Paternal education (secondary+ vs. none)	0.064	-0.002	0.130	0.059	20.9
**Covariates**	**0.004**	**-0.051**	**0.060**	**0.874**	**1.5**
Child sex (female vs. male)	-0.009	-0.019	0.000	0.057	-3.1
Child age (12-23 mo vs. 6-11 mo)	0.015	-0.011	0.041	0.263	4.9
Birth order (2-4 vs. 1st)	0.006	-0.005	0.017	0.300	1.9
Birth order (≥5 vs. 1st)	-0.021	-0.051	0.008	0.156	-7.0
Sex of household head (female vs. male)	-0.005	-0.018	0.007	0.396	-1.8
Size at birth (small vs. average)	0.023	0.007	0.038	0.004	7.4
Size at birth (large vs. average)	-0.007	-0.017	0.003	0.187	-2.2
Problems accessing care (yes vs. no)	-0.016	-0.041	0.008	0.195	-5.4
Minimum dietary diversity (achieved vs. not achieved)	0.021	0.001	0.040	0.042	6.7
**Country fixed effects**	**-0.074**	**-0.114**	**-0.033**	**<0.001**	**-24.1**
**Total explained gap**	0.305	0.194	0.416	<0.001	100.0
**Total unexplained gap**	0.001	-0.134	0.135	0.994	-
**Total predicted gap**	0.306	0.195	0.417	<0.001	-

STROBE Statement—Checklist of items that should be included in reports of *cross-sectional studies*

	Item No	Recommendation	Page
**Title and abstract**	1	(*a*) Indicate the study's design with a commonly used term in the title or the abstract	*p.1*
		(*b*) Provide in the abstract an informative and balanced summary of what was done and what was found	*pp.1-2*
**Introduction**			
Background/rationale	2	Explain the scientific background and rationale for the investigation being reported	*pp.2-3*
Objectives	3	State specific objectives, including any prespecified hypotheses	*p.4*
**Methods**			
Study design	4	Present key elements of study design early in the paper	*p.4*
Setting	5	Describe the setting, locations, and relevant dates, including periods of recruitment, exposure, follow-up, and data collection	*p.4*
Participants	6	(*a*) Give the eligibility criteria, and the sources and methods of selection of participants	*pp.4-5*
Variables	7	Clearly define all outcomes, exposures, predictors, potential confounders, and effect modifiers. Give diagnostic criteria, if applicable	*pp.5-6*
Data sources/ measurement	8*	For each variable of interest, give sources of data and details of methods of assessment (measurement). Describe comparability of assessment methods if there is more than one group	*pp.4-7*
Bias	9	Describe any efforts to address potential sources of bias	*pp.7-8*
Study size	10	Explain how the study size was arrived at	*p.4*
Quantitative variables	11	Explain how quantitative variables were handled in the analyses. If applicable, describe which groupings were chosen and why	*pp.5-7*
Statistical methods	12	(*a*) Describe all statistical methods, including those used to control for confounding	*p.7-8*
		(*b*) Describe any methods used to examine subgroups and interactions	*p.8*
		(*c*) Explain how missing data were addressed	*p.7*
		(*d*) If applicable, describe analytical methods taking account of sampling strategy	*p.7*
		(*e*) Describe any sensitivity analyses	*p.8*
**Results**			
Participants	13*	(a) Report numbers of individuals at each stage of study—eg numbers potentially eligible, examined for eligibility, confirmed eligible, included in the study, completing follow-up, and analysed	*P.9*
		(b) Give reasons for non-participation at each stage	*NA*
		(c) Consider use of a flow diagram	*NA*
Descriptive data	14*	(a) Give characteristics of study participants (eg demographic, clinical, social) and information on exposures and potential confounders	*P.9*
		(b) Indicate number of participants with missing data for each variable of interest	*Table 1*
Outcome data	15*	Report numbers of outcome events or summary measures	*p.9*
Main results	16	(*a*) Give unadjusted estimates and, if applicable, confounder-adjusted estimates and their precision (eg, 95% confidence interval). Make clear which confounders were adjusted for and why they were included	*pp.9-11*
		(*b*) Report category boundaries when continuous variables were categorized	*pp.9-10*
		(*c*) If relevant, consider translating estimates of relative risk into absolute risk for a meaningful time period	*NA*

Other analyses	17	Report other analyses done—eg analyses of subgroups and interactions, and sensitivity analyses	*p.11*
**Discussion**			
Key results	18	Summarise key results with reference to study objectives	*p.11*
Limitations	19	Discuss limitations of the study, taking into account sources of potential bias or imprecision. Discuss both direction and magnitude of any potential bias	*pp.13-14*
Interpretation	20	Give a cautious overall interpretation of results considering objectives, limitations, multiplicity of analyses, results from similar studies, and other relevant evidence	*pp.11-14*
Generalisability	21	Discuss the generalisability (external validity) of the study results	*p.14*
**Other information**			
Funding	22	Give the source of funding and the role of the funders for the present study and, if applicable, for the original study on which the present article is based	*p.15*

*Give information separately for exposed and unexposed groups.

Note: An Explanation and Elaboration article discusses each checklist item and gives methodological background and published examples of transparent reporting. The STROBE checklist is best used in conjunction with this article (freely available on the Web sites of PLoS Medicine at http://www.plosmedicine.org/, Annals of Internal Medicine at http://www.annals.org/, and Epidemiology at http://www.epidem.com/). Information on the STROBE Initiative is available at www.strobe-statement.org.
